# The impact of undergraduate nursing students’ time management disposition on innovative behavior: the chain mediating role of academic self-efficacy and flow experience

**DOI:** 10.3389/fpsyg.2025.1447121

**Published:** 2025-02-19

**Authors:** Ye Lin, Xixi Wang, Xinyi Zhang, Hongli Hu, Li Liu, Keyi Pang, Yan Li, Chaoqun Hu, Zhiqin Sun, Xiaona Li

**Affiliations:** ^1^School of Medicine and Health Engineering, Changzhou University, Changzhou, China; ^2^Department of Health Service Support, Naval Medical University, Shanghai, China; ^3^Department of Nursing, The Second People’s Hospital of Changzhou, the Third Affliated Hospital of Nanjing Medical University, Changzhou, China

**Keywords:** time management disposition, innovative behavior, academic self-efficacy, flow experience, chain mediation, undergraduate nursing students

## Abstract

**Objective:**

To explore the chain intermediary effect of academic self-efficacy and flow experience of undergraduate nursing students in the influence mechanism of time management disposition on innovative behavior.

**Methods:**

Seven hundred and sixty-three undergraduate nursing students from 3 universities in Jiangsu Province were investigated with Adolescent time Management Disposition Scale, Academic Self-efficacy Scale, Flow Short Scale and Innovation Behavior Scale. The chain intermediary model was constructed and verified.

**Results:**

The time management disposition of undergraduate nursing students was positively correlated with their average scores in academic self-efficacy, flow experience, and innovative behavior. The direct impact of time management disposition on innovative behavior was significant, and academic self-efficacy and flow experience exhibited both independent mediating effects and a chain mediating effect.

**Conclusion:**

The time management disposition of undergraduate nursing students can significantly positively predict innovative behavior, in which academic self-efficacy and flow experience play a significant independent intermediary role and chain intermediary role. Nursing teachers and students can promote innovative behavior from the point of view of improving time management skills, enhancing academic self-efficacy and flow experience.

## 1 Introduction

Given the rapid development of the social economy and the changes in population structure, there is a dual trend of diversification and increased quality in the demand for healthcare services among the public. This necessitates continuous exploration of innovative pathways within the nursing field to effectively address the increasingly complex health challenges. Innovative behavior, defined as the process in which individuals actively adopt and implement novel ideas, frontier knowledge, or unique solutions in innovative activities (Amabile, 1988), not only reflects personal ability and creativity but is also a key driver in advancing the discipline of nursing and enhancing the quality of nursing services. Undergraduate nursing students, as the backbone and potential reserve force of the future nursing field, play a crucial role in the cultivation and practice of innovative capabilities, which is essential for raising the professional level of nursing services, optimizing the patient care experience, and leading the forefront of nursing science exploration. They are not only tasked with the mission of inheriting and applying existing nursing knowledge and practical skills but also bear the responsibility of innovating nursing theories and developing new nursing techniques and methods. Therefore, the innovative behavior of undergraduate nursing students is not only relevant to individual professional growth and skill enhancement but also has a profound impact on the overall progress of the nursing discipline and the improvement of societal well-being. However, in the face of numerous challenges in higher nursing education for cultivating innovative nursing talent, effectively stimulating students’ innovative consciousness and cultivating their ability to translate theoretical knowledge and practical experience into tangible innovative outcomes has become a critical issue that demands immediate resolution (Xiang et al., 2023). Against this backdrop, the present study focuses on the innovative behavior of undergraduate nursing students and delves into the underlying influencing factors and mechanisms.

Previous studies have shown that individual innovative behavior is influenced by a variety of factors, including personality traits (such as creativity, proactivity, and initiative personality traits) (Abdullah et al., 2016; Dai et al., 2024), motivational factors (such as intrinsic motivation and extrinsic rewards) (Karadeniz, 2021; Yaqoob and Kitchlew, 2022), and organizational environment (such as organizational culture and leadership support) (Çalışkan et al., 2014; Mokhber et al., 2018). Although the field of innovative behavior has accumulated substantial valuable research, few researchers have focused on the impact of time management disposition, an aspect of personality related to time, on innovative behavior. Time management disposition refers to the attitudes, habits, and behavioral patterns that individuals exhibit towards time in their daily lives. It reflects an individual’s psychological characteristics and abilities in planning, allocating, and utilizing time resources to achieve personal goals and improve the efficiency of life. This disposition typically includes three components: time value perception, time monitoring perspective, and time efficacy perception (Huang and Zhang, 2001). As a stable personality trait exhibited by individuals in time and task management, time management disposition profoundly affects the strategic choices, mental adjustments, and resource allocation of individuals when facing innovative challenges. Therefore, time management disposition is likely to be associated with innovative behavior.

Existing literature indicates that nursing students often face issues with poor time management in their studies and internships, which primarily stem from factors such as heavy course loads, compact clinical practice schedules, and insufficient personal self-management skills (Mirzaei et al., 2012; Zhang et al., 2021). Studies also suggest that effective time management can not only improve learning efficiency but also enhance students’ professional quality and mental health, as well as affect their ability to provide high-quality care to patients (Mirzaei et al., 2012; Behdarvand et al., 2023; Çingöl and Karakaş, 2023). The innovative behavior of nursing students is generally at a moderate level and shows significant differences among individuals (Xiang et al., 2023). Some nursing students may exhibit a higher degree of innovative enthusiasm, actively exploring and implementing new ideas, while others may be more conservative and passive in demonstrating innovative behavior (Zhong et al., 2018). Therefore, in a situation where both time management skills and innovative behavior of undergraduate nursing students face challenges and opportunities, investigating the impact of time management disposition on innovative behavior holds important practical significance for improving the overall quality of nursing students and the quality of nursing services.

## 2 Theory and hypothesis

### 2.1 The influence of time management disposition on innovative behavior

The conservation of resources theory suggests that individuals are inclined to acquire, protect, and maintain resources to cope with the stresses and challenges of life (Bon and Mohamud, 2022). Time is a critical resource, and time management disposition directly affects the allocation and efficiency of an individual’s time resources. Individuals with a high disposition for time management can allocate their time resources more effectively, thus having more time and energy to invest in innovative activities, which provide a resource guarantee for the emergence of innovative behavior. The self-regulation theory emphasizes that individuals achieve their goals through self-regulatory processes (Mann et al., 2013). Time management disposition, as an important aspect of self-regulation, can help individuals set clear goals, plan the timeline for innovative activities, monitor the innovation process, and adjust strategies as needed. This self-regulatory ability is an important prerequisite for the occurrence of innovative behavior. Studies (Zampetakis et al., 2010; Darini et al., 2011) have shown a positive correlation between individuals’ confidence in time planning and their creativity and innovation. Further research indicated that individuals possessing advanced time control skills are advantageous in enhancing creativity (Van Eerde et al., 2022). Creativity and innovative behavior are two interconnected yet distinct concepts. Creativity is the foundation of innovative behavior, referring to an individual’s ability to generate novel and valuable ideas, while innovative behavior involved the transformation of creative thinking from intangible to tangible, with a focus on the execution process (Ishiguro et al., 2022). Existing research had confirmed that an individual’s creativity was an important indicator for predicting their innovative behavior. Individuals with high creativity were more likely to take action and translate their ideas into concrete innovative outcomes (Mittone et al., 2022). Therefore, this study proposes the first hypothesis (H1): The time management disposition of undergraduate nursing students could positively predict innovative behavior.

### 2.2 The mediating role of academic self-efficacy

The self-efficacy theory, proposed by Bandura (1978), posited that self-efficacy was an individual’s belief in their ability to execute specific tasks, which directly influenced their behavior choices, effort levels, persistence, and emotional state when facing tasks. Academic self-efficacy is the manifestation of self-efficacy in the academic domain (Bowman et al., 2019). Individuals with a high disposition for time management are often able to effectively plan and allocate their study time, thereby improving both the efficiency and quality of task completion. This dual enhancement of efficiency and quality, in turn, bolsters their confidence in their academic abilities, ultimately leading to an increase in academic self-efficacy (Bargmann and Kauffeld, 2023). Academic self-efficacy can strengthen students’ intrinsic motivation, prompting them to actively explore new knowledge and methods, which in turn stimulates creativity (Capron Puozzo and Audrin, 2021). Studies have shown that students with high self-efficacy are more willing to try different solutions in their learning, demonstrating stronger innovative thinking (Zhang, 2023). Additionally, academic self-efficacy is closely related to an individual’s goal setting. Students with high self-efficacy tend to set more challenging goals, and this goal orientation can motivate them to continuously make breakthroughs in innovative behavior (Saks, 2024). Positive academic self-efficacy enhances students’ psychological resilience, enabling them to recover more quickly from failures and continue with innovative attempts (Cassidy, 2015). This emotional stability provides a solid psychological foundation for innovative behavior. Empirical research has indicated that college students’ time management skills are directly related to self-efficacy, and improvements in time management are beneficial for enhancing self-efficacy (Sevari and Kandy, 2011; Bargmann and Kauffeld, 2023; Liu and Lu, 2024). Typically, individuals with strong self-efficacy tend to possess pioneering innovative thinking. Conversely, a decrease in self-efficacy can hinder innovative behavior (Zhang, 2023). Based on this, the study proposes the second hypothesis (H2): Academic self-efficacy of undergraduate nursing students plays a mediating role in the relationship between time management disposition and innovative behavior.

### 2.3 The mediating role of flow experience

The theory of flow was proposed by psychologist Csíkszentmihályi (1975), which described a state of immersion that individuals experienced when they were fully engaged and enjoying an activity, known as the flow experience. This state is characterized by heightened concentration, loss of self-consciousness, a profound sense of engagement with the activity, and a distorted sense of time. Individuals with a high disposition for time management are capable of creating an environment conducive to flow experiences, such as reducing distractions, setting clear goals, and providing timely feedback, all of which are key elements of the flow experience. According to the flow theory, when individuals experience flow in an activity, they are inclined to break routines, exhibit divergent and creative thinking, and actively explore new things, thereby facilitating the emergence of innovative behaviors. The heightened focus and enjoyment associated with the flow state facilitate the generation of new ideas and the resolution of problems. A survey conducted in Japan indicated that university students with strong time management skills experience more flow in their daily activities (Ishimura and Kodama, 2009). Chinese scholars have also found a positive correlation between students’ time management disposition and flow experience (Zhang, 2019). Multiple studies have confirmed that flow experiences contribute to the generation of high-level creativity and innovative behaviors (Primus and Sonnenburg, 2018; Zhou et al., 2021; Deng et al., 2022). Therefore, this study proposes the third hypothesis (H3): the flow experience of undergraduate nursing students mediates the relationship between time management disposition and innovative behavior.

### 2.4 The chain mediating role of academic self-efficacy and flow experience

Self-efficacy not only serves as a significant predictor of individual innovative behavior but is also closely related to the flow experience. According to the flow theory, flow can be characterized by its antecedents, the flow experience itself, and its consequences. Self-efficacy is a crucial component of the antecedents of flow (Barthelmäs and Keller, 2021). Social cognitive theory posits that self-efficacy is an individual’s belief in their ability to execute specific tasks, which has a profound impact on their behavior, motivation, and emotional responses when facing challenges (Cook and Artino, 2016). When individuals possess high academic self-efficacy, they are more likely to choose academic tasks with an appropriate level of challenge and approach them with a positive attitude and actions, aligning with the flow theory’s perspective on the balance between challenge and skill. Academic self-efficacy can also reduce the sense of anxiety and stress individuals experience during academic pursuits (Kaufmann et al., 2022), enabling them to focus more intently on the task at hand, thereby increasing the likelihood of experiencing flow. Empirical research has shown that the self-efficacy generated by college students during their learning process can facilitate their experience of flow (Joo et al., 2015; Cai and Jia, 2020). Therefore, this study proposes the fourth hypothesis (H4): The academic self-efficacy and flow experience of undergraduate nursing students play a chain mediating role between time management disposition and innovative behavior.

## 3 Methodology

### 3.1 Object and data collection

Utilizing convenience sampling, this study selected undergraduate nursing students from three universities in Jiangsu Province, ranging from freshmen to seniors, as the research subjects. Prior to the survey, the content and purpose of the investigation were explained, and informed consent was obtained from the nursing students. The questionnaires were then distributed by trained surveyors using the questionnaire Wenjuanxing platform.^1^ To control for potential common method bias in this study, measures such as anonymity and reverse scoring for some items were implemented during the survey administration process. The data collection period spanned from June 2023 to December 2023. A total of 800 questionnaires were distributed. An initial inspection was conducted on all collected questionnaires to exclude invalid ones. The criteria for determining invalid questionnaires included: incomplete surveys, answers that displayed obvious patterns (e.g., an excessive number of the same options selected), and excessively short response times (less than half of the average completion time). Ultimately, 763 valid questionnaires were collected, resulting in an effective response rate of 95.38%.

### 3.2 Measures

#### 3.2.1 Time management disposition

The study employed the Adolescent Time Management Disposition Scale developed by Huang and Zhang (2001). This scale consists of three dimensions: time value, time monitoring, and time efficiency, comprising a total of 44 items (items 9, 17, 27, 30, and 41 are reverse-scored), and uses a Likert 5-point rating scale (1 = completely disagree, 5 = completely agree). A higher score indicates a stronger time management disposition. Example items: 1. I believe that the saying “Time is money” is correct. (A. Completely Disagree, B. Somewhat Disagree, C. Neutral, D. Somewhat Agree, E. Completely Agree). 2. I usually organize my daily activities into a schedule (A. Completely Disagree, B. Somewhat Disagree, C. Neutral, D. Somewhat Agree, E. Completely Agree). In this study, the Cronbach’s alpha coefficient for this scale was 0.944.

#### 3.2.2 Academic self-efficacy

The study utilized the Academic Self-Efficacy Scale revised by Lee et al. (2010) This scale includes 7 items (all positively scored), using a Likert 5-point rating scale (1 = completely disagree, 5 = completely agree). A higher score indicates a stronger academic self-efficacy among students. Previous research has confirmed the scale’s reliability and validity (Han, 2021). Example items: 1. I believe I can understand the most difficult parts taught by the teacher in class. (A. Completely Disagree, B. Somewhat Disagree, C. Neutral, D. Somewhat Agree, E. Completely Agree). 2.I believe I can grasp the basic concepts taught by the teacher in class (A. Completely Disagree, B. Somewhat Disagree, C. Neutral, D. Somewhat Agree, E. Completely Agree). In this study, the Cronbach’s alpha coefficient for this scale was 0.877.

#### 3.2.3 Flow experience

The study employed the Flow Short Scale (FKS) designed by Klauer et al. (2004) This scale consists of 13 items (all positively scored), using a Likert 7-point rating scale (1 = Strongly disagree, 7 = Strongly agree). A higher score indicates a more frequent flow experience among students. Previous research has confirmed the scale’s reliability and validity (Jiang and Jiang, 2015). Example items: 1. I am brave in trying new things when I study (A. Strongly disagree, B. Disagree, C. Slightly disagree, D. Neutral, E. Slightly agree, F. Agree, G. Strongly agree). 2. The tasks I challenge are at the same level as my own skills (A. Strongly disagree, B. Disagree, C. Slightly disagree, D. Neutral, E. Slightly agree, F. Agree, G. Strongly agree.) In this study, the Cronbach’s alpha coefficient for this scale was 0.933.

#### 3.2.4 Innovative behavior

The study utilized the Innovative Behavior Scale developed by Liu and Shi (2009). This scale includes 5 items (all positively scored), using a Likert 7-point rating scale (1 = Strongly disagree, 7 = Strongly agree). A higher score indicates a greater frequency of innovative behavior among students. Example items: 1. During the learning process, I often have new ideas and perspectives. (A. Strongly disagree, B. Disagree, C. Slightly disagree, D. Neutral, E. Slightly agree, F. Agree, G. Strongly agree). 2. I can present my new idea to my classmates or teachers to gain their support and recognition. (A. Strongly disagree, B. Disagree, C. Slightly disagree, D. Neutral, E. Slightly agree, F. Agree, G. Strongly agree). In this study, the Cronbach’s alpha coefficient for this scale was 0.854.

### 3.3 Statistical analysis

This study employed SPSS 24.0 and AMOS 26.0 statistical software for data analysis. Firstly, the study explored the factors influencing innovative behavior and the correlation between time management disposition, academic self-efficacy, flow experience, and innovative behavior. To achieve this, descriptive statistics were conducted using SPSS 24.0, with normally distributed measurement data described by mean ± standard deviation ( $\bar{x}$ **±s**), and categorical data described by frequency and percentage. Univariate analysis was performed using independent sample t-tests and analysis of variance (ANOVA), while correlation analysis was conducted using Spearman’s correlation test. The common method bias test was carried out using the Harman single-factor method. Subsequently, the AMOS 26.0 statistical software was employed to test the chain mediating effects. Model fit was assessed using fit indices such as χ2/df, RMR, RMESA, CFI, GFI, AGFI, TLI, and IFI. A model is considered to fit well if χ2/df < 3, RMR < 0.05, RMESA <0.08, and CFI, GFI, AGFI, TLI, and IFI > 0.9. Bootstrap resampling was conducted 5,000 times, and a 95% confidence interval (CI) was calculated. A confidence interval not including zero indicates a significant effect. A *p*-value <0.05 indicates a statistically significant difference.

## 4 Results

### 4.1 Common method bias test

The Harman single-factor test was employed to examine the common method bias in the survey data. The results indicated that there was a total of 10 factors with eigenvalues greater than 1. The first factor accounted for 34.50% of the variance, which was less than the critical threshold of 40%, suggesting that there was no severe common method bias present.

### 4.2 Univariate analysis of innovative behavior

The ages of the 763 undergraduate nursing students ranged from 17 to 22 years (19.55 ± 1.20 years). There were 197 (25.8%) first-year students, 168 (22.0%) second-year students, 218 (28.6%) third-year students, and 180 (23.6%) fourth-year students. In terms of gender, there were 651 females (85.3%) and 112 males (14.7%). Regarding their place of residence, 285 students (37.4%) came from rural areas, 214 (28.0%) from towns, and 264 (34.6%) from cities. In terms of whether they were only children, 457 students (59.9%) were not, while 306 (40.1%) were. Regarding their college entrance examination volunteer selection, 564 students (73.9%) made their choices voluntarily, 70 (9.2%) were required by their parents to choose, and 129 (16.9%) were placed through adjustment. During their time at the university, 335 students (43.9%) had received 0 awards, 285 (37.4%) had received 1–3 awards, 72 (9.4%) had received 4–5 awards, and 71 (9.3%) had received 6 or more awards.

As shown in Table 1, the gender and the receipt of awards during the academic tenure were significant factors influencing the scores of innovative behavior among undergraduate nursing students (*p* < 0.01).

**Table 1:** Univariate analysis of innovative behavior among undergraduate nursing students.

Items	Cases [*N*(%)]	Innovative behavior scores ( $\bar{x}$ ±s)	t/F	*p*
Grade			0.669	0.571
First-year	197 (25.8)	4.42 ± 1.01		
Second-year	168 (22.0)	4.33 ± 1.04		
Third-year	218 (28.6)	4.30 ± 0.95		
Fourth-year	180 (23.6)	4.42 ± 1.00		
Gender			−3.351	0.001^**^
Female	651 (85.3)	4.32 ± 0.98		
Male	112 (14.7)	4.66 ± 1.06		
Place of residence			0.551	0.577
Rural	285 (37.4)	4.34 ± 0.98		
Town	214 (28.0)	4.33 ± 0.95		
City	264 (34.6)	4.42 ± 1.05		
Only child			−0.348	0.728
No	457 (59.9)	4.36 ± 1.01		
Yes	306 (40.1)	4.38 ± 0.98		
College entrance examination volunteer selection			1.837	0.160
Self-chosen	564 (73.9)	4.41 ± 1.00		
Parental requirement	70 (9.2)	4.21 ± 1.03		
Adjustment	129 (16.9)	4.28 ± 0.95		
Awards received			4.190	0.006^***^
0 items	335 (43.9)	4.29 ± 1.00		
1–3 items	285 (37.4)	4.33 ± 0.99		
4–5 items items	72 (9.4)	4.52 ± 0.93		
6 or more items	71 (9.3)	4.71 ± 1.01		

**p < 0.01, ***p < 0.001.

### 4.3 Means, standard deviations, and correlation analysis among variables

As shown in Table 2, there were significant positive correlations between undergraduate nursing students’ time management disposition and academic self-efficacy, flow experience, and innovative behavior. Additionally, academic self-efficacy was significantly positively correlated with flow experience and innovative behavior. Furthermore, flow experience and innovative behavior were significantly positively correlated.

**Table 2:** The descriptive statistics and correlation analysis among variables.

	Mean Item Score ( $\bar{x}$ ±s)	TMD	ASE	FE	IB
TMD	3.40 ± 0.49	1			
ASE	3.42 ± 0.65	0.691**	1		
FE	4.36 ± 0.99	0.732**	0.750**	1	
IB	4.37 ± 1.00	0.630**	0.617**	0.694**	1

**p < 0.01. TMD, time management disposition; ASE, academic self-efficacy; FE, flow experience; IB, innovative behavior.

### 4.4 Chain mediation test of academic self-efficacy and flow experience

**Figure 1:**
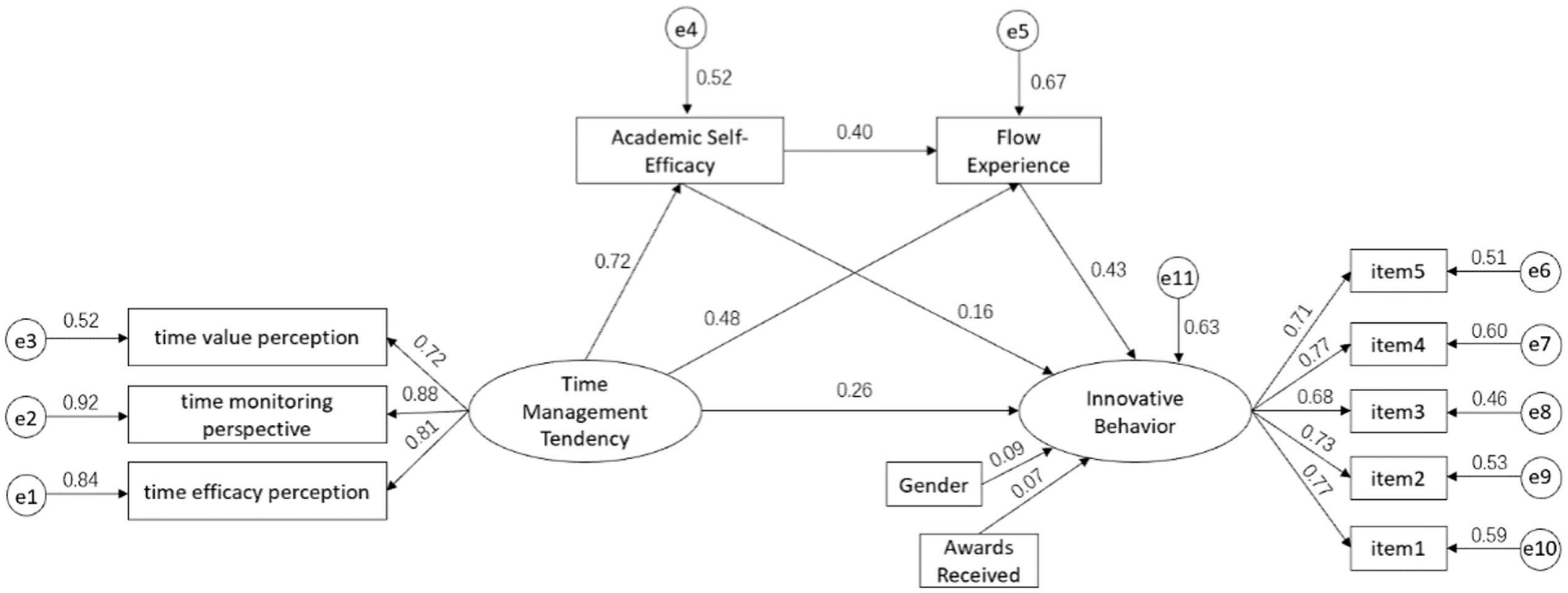
The relationship between time management disposition and innovative behavior: a chain mediation effect model of academic self-efficacy and flow experience.

After controlling for the variables of gender and awards received during the academic tenure, a structural equation model was constructed with time management disposition as the independent variable, innovative behavior as the dependent variable, and academic self-efficacy and flow experience as mediating variables. The results indicated that the model fit the data well, with fit indices of χ2/df = 2.870, RMR = 0.031, RMSEA = 0.050, CFI = 0.982, GFI = 0.971, AGFI = 0.954, TLI = 0.976, and IFI = 0.982. The model and standardized path coefficients were presented in Figure 1.

The results of the Bootstrap analysis revealed that the direct effect of time management disposition on innovative behavior was significant (effect size 0.263). Both academic self-efficacy (effect size 0.115, 95% CI [0.037, 0.191]) and flow experience (effect size 0.205, 95% CI [0.149, 0.265]) independently mediated the relationship between time management disposition and innovative behavior. Additionally, the chain mediating effect of academic self-efficacy and flow experience on the influence of time management disposition on innovative behavior was significant (effect size 0.126, 95% CI [0.087, 0.173]) (Table 3).

**Table 3:** Bootstrap test of the mediation effects between time management disposition and innovative behavior.

Item	Effect size	SE	95%CI	Proportion of effect (%)
TMD → ASE → IB	0.115	0.039	0.037–0.191	16.2
TMD → FE → IB	0.205	0.029	0.149–0.265	28.9
TMD → ASE → FE → IB	0.126	0.022	0.087–0.173	17.8
Total mediation effect	0.446	0.044	0.364–0.536	62.9
Direct effect	0.263			37.1

TMD, time management disposition; ASE, academic self-efficacy; FE, flow experience; IB, innovative behavior.

## 5 Discussion

### 5.1 Undergraduate nursing students’ innovative behaviors are at a moderate level

This study found that the mean score of undergraduate nursing students’ innovative behaviors was (4.37 ± 1.00), indicating a moderate level, consistent with the findings of Zhou et al. (2018). In recent years, entrepreneurship and innovation education in Chinese universities has been thriving. Most universities are able to foster a conducive atmosphere for innovation, encouraging students’ innovative behaviors. This is a significant reason for the improvement in undergraduate nursing students’ innovative behaviors compared to a decade ago (Zhao et al., 2011). However, despite innovation becoming a hot topic in nursing education, the integration of innovative knowledge, skills, and attitudes into undergraduate nursing curricula is still in its infancy. There is still considerable room for improvement in the innovative abilities of undergraduate nursing students. Nursing educators need to actively cultivate an innovative atmosphere, integrating the cultivation of students’ innovative behaviors with teaching content. They should guide students to identify potential problems in the field of nursing, encourage them to think boldly, and propose more novel ideas, thereby stimulating the generation of more innovative behaviors.

### 5.2 Undergraduate nursing students’ time management disposition positively predicts innovative behaviors

Innovation behavior is influenced by various personality traits. However, few researchers have focused on the influence of personality traits in the temporal dimension on innovative behaviors. This study examined the relationship between undergraduate nursing students’ time management disposition and innovative behaviors. The results indicated that time management disposition significantly positively predict innovative behaviors, supporting hypothesis H1. In recent years, research has shown that the transformation of creativity into innovative behavior requires effective self-regulation processes (Ivcevic et al., 2023). Time management is the application of self-regulation processes in the temporal domain. Self-regulation theory divides the self-regulation process into two stages: goal setting and goal implementation (Mann et al., 2013). Nursing students with strong time management skills can overcome obstacles in the innovation process through activities such as setting innovative goals, developing planning strategies, scheduling time effectively, and providing feedback on outcomes, ultimately facilitating the realization of creative ideas.

### 5.3 The mediating role of academic self-efficacy in the relationship between time management disposition and innovative behavior among undergraduate nursing students

This study found that the positive predictive effect of undergraduate nursing students’ time management disposition on innovative behaviors could be achieved both through a direct pathway and by increasing academic self-efficacy, indirectly linking to innovative behaviors, supporting hypothesis H2. Nursing students with strong time values and monitoring perspectives can reduce procrastination and enhance efficiency in learning, which contributes to achieving good academic performance and increasing self-efficacy (Nasrullah and Saqib Khan, 2015). This study also confirmed the importance of self-efficacy in promoting individual creative thinking and creativity (Orakci, 2023; Zhang, 2023; Yao et al., 2024). The reason lies in the fact that students with higher academic self-efficacy are more motivated and confident in thinking, discussing, and actively facing challenges, which helps them pursue and manage the innovation process with greater perseverance in handling innovative problems (Royston and Reiter-Palmon, 2019). Conversely, students with low academic self-efficacy lack motivation and struggle to address innovative technical issues and challenges, participating less in innovative activities or even adopting a resistant attitude (Zhang, 2023). Additionally, students inevitably encounter various difficulties and pressures in innovative behaviors. In adverse situations, the positive supportive role of academic self-efficacy in innovative behaviors becomes particularly prominent (Travis et al., 2020).

### 5.4 The mediating role of flow experience in the relationship between time management disposition and innovative behavior among undergraduate nursing students

This study found that the time management disposition of undergraduate nursing students can be linked to innovative behavior through the enhancement of flow experience, confirming hypothesis H3. Novak et al. (2000) conceptualized the essence of flow experience into three factors based on its developmental process: conditional factors (balance of challenge and skill, clear goals, immediate feedback), experiential factors (deep concentration, fusion of action and awareness, sense of control), and consequential factors (time distortion, loss of self-consciousness, physical sensation). The time management disposition of nursing students can facilitate the achievement of flow’s conditional factors. Good time management skills not only help students set goals that match their abilities but also drive them to act towards specific goals and provide active feedback during action (Huang and Zhang, 2001). The time management disposition of nursing students can also directly promote the achievement of flow’s experiential factors. Students with strong time management skills can allocate their time according to their circadian rhythms, scheduling high-focus learning during the brain’s golden hours, thus ensuring a high level of concentration (Dacoylo et al., 2024). The flow results induced by nursing students’ flow experience primarily include the generation of positive emotions (Mulik et al., 2019) and the improvement of academic performance (Mulik et al., 2019), which help students develop a strong interest in new methods and knowledge, form flexible and open innovative thinking, and invest great passion into innovative practices, undaunted by the challenges and setbacks in the innovation process (Hakanen et al., 2008). Additionally, according to the extension-construction theory, the flow results of nursing students can also increase the quantity and breadth of resources connected to problem-solving by expanding their cognitive scope and dispersing attention, and enable them to reconstruct their action command system, pursuing creative thoughts and action paths (Zhou et al., 2020).

### 5.5 The chain mediating role of academic self-efficacy and flow experience between time management disposition and innovative behavior in undergraduate nursing students

The results of this study also indicated that academic self-efficacy and flow experience in undergraduate nursing students played a chain mediating role between time management disposition and innovative behavior, supporting hypothesis H4. Multiple studies have suggested that there may be a complementary and mutually reinforcing positive correlation between academic self-efficacy and flow experience. In this study, the self-efficacy of undergraduate nursing students significantly and positively predicted flow experience, consistent with the findings of Joo et al. (2015) and Cai and Jia (2020). Nursing students with high self-efficacy are more likely to actively set challenging goals and experience a strong sense of time distortion while overcoming challenges and achieving goals, thus frequently entering a state of flow. However, researches also indicate that flow experience can have an important positive impact on self-efficacy (Mao et al., 2020; Wu et al., 2021). Students with high levels of flow experience find it easier to develop a stable sense of control while completing academic tasks, effectively mobilizing their cognitive and skill resources to meet these tasks, thereby experiencing a strong sense of immersion and enjoyment. These positive experiences can enhance their confidence in achieving learning goals and promote the enhancement of academic self-efficacy (Wei and Chen, 2022). Conversely, students with lower levels of flow experience may struggle to handle tasks that do not match their skills, fail to take proactive actions in the face of academic challenges, and are prone to experiencing setbacks in academic activities, leading to lower levels of self-efficacy.

## 6 Research significance and limitations

This study, employing a quantitative research method, explores the impact of undergraduate nursing students’ time management disposition on innovative behavior and the chain mediating effects of academic self-efficacy and flow experience within this context, which holds theoretical and practical significance. Theoretically, this study reveals the complex relationships among time management disposition, academic self-efficacy, flow experience, and innovative behavior, providing a theoretical foundation for future research. Additionally, through the exploration of chain mediation effects, it deepens the understanding of the mechanisms underlying innovative behavior. Furthermore, the methodology employed in this study serves as a reference for similar research, contributing to the advancement of quantitative research in the field of nursing education at a deeper level. Practically, it provides a new perspective for the research on strategies for cultivating outstanding innovative talents in nursing. First, due to the fact that time management disposition promotes academic self-efficacy, flow experience, and innovative behavior, nursing faculty can introduce concepts and strategies of time management in their daily teaching or by offering time management courses and seminars to help nursing students manage and optimize their time effectively, thereby enhancing personal efficiency. Additionally, teaching models should shift from a teacher-centered passive mode to a student-centered active mode to facilitate nursing students’ better time management, effective learning, and improved academic performance. Second, nursing faculty can employ various methods to enhance nursing students’ self-efficacy. For example, attributing academic success or failure to effort rather than ability; providing opportunities for peer observation to strengthen nursing students’ belief in their ability to achieve good results; ensuring nursing students receive support from reliable sources (such as teachers)(Zheng et al., 2021). Third, nursing faculty should focus on stimulating students’ flow experiences during the teaching process. For instance, designing various interesting and challenging tasks and activities for nursing students to choose from, setting clear problem-solving goals to ensure their focus; allowing nursing students to complete tasks progressively from easy to difficult to achieve a balance between challenge and skill; and providing timely feedback on task progress and rewards accordingly.

There are several limitations to this study. First, it is a cross-sectional survey and cannot accurately infer causality. Subsequent research can delve deeper into the impact of undergraduate nursing students’ time management disposition on innovative behavior through experimental interventions or longitudinal research. Second, the study only selected undergraduate nursing students as the research subjects, and the results should be used with caution when generalized to other groups. Third, the mediating effects of self-efficacy and flow experience are incomplete, indicating that further exploration of the impact mechanism of time management disposition on innovative behavior should consider other influencing factors. In future research, it would be valuable to further explore the differences in time management disposition and innovative behaviors among undergraduate nursing students within cross-cultural contexts, as well as to examine the trends of change and the impact of individual differences over long-term tracking of personal development. These directions not only contribute to deepening theoretical understanding and refining existing models but also provide more precise and personalized strategies for nursing education practice.

## Data Availability

The raw data supporting the conclusions of this article will be made available by the authors, without undue reservation.

## Footnotes

^1^ https://www.wjx.cn/
